# Stafne's Bone Defect Correlated with Submandibular Glands: A Case Report and CBCT and MRI Analysis

**DOI:** 10.1155/2024/1173783

**Published:** 2024-02-06

**Authors:** Antoine Berberi

**Affiliations:** Faculty of Dental Medicine, Lebanese University, Beirut, Lebanon

## Abstract

Stafne's bone defect is a developmental anatomic bone defect in the lingual side of the mandible in the area of the mandibular angle that is filled with proliferation or translocation of adjacent structures such as salivary gland tissue. The etiology is still undefined, and two hypotheses are proposed: one is the glandular related to the submandibular or sublingual glands and the second is ischemic that affects the vascularization of the mandibular lingual. Usually, Stafne's bone defect is accidentally detected on panoramic radiographs during dental treatments as a well-limited radiolucency image with a clear peripheral regular condensation border, located below the mandibular canal. The differential diagnosis includes traumatic bone cyst, odontogenic and nonodontogenic cystic lesions, nonossifying fibroma, focal osteoporotic bone marrow defect, and other lesions. A case of Stafne's bone defect on a 60-year-old male patient extending in the lingual posterior part of the mandibular region was presented. The panoramic radiograph revealed a well-limited radiolucency image with a clear peripheral regular condensation border, located below the mandibular canal. The lesion was discovered in a routine radiographic exam: the cone beam computed tomography gave us more details about the localization, the shape and size, and the relation with the mandibular canal, and the magnetic resonance imaging identifies the nature of the inside soft tissue. The final diagnosis was a Stafne's bone defect resulting of a depression of the lingual cortical plate filled with expansion of the submandibular gland.

## 1. Introduction

Stafne's bone defect (SBD) was first reported in 1942 by Edward Stafne as radiolucent lesions in the mandibular angle discovered on intraoral films [1].

SBD is considered as a developmental anatomic bone defect localized in the lingual part of the mandible and is occupied with proliferation or translocation of adjacent structures such as salivary gland tissue, adipose tissue, and lymphatic tissue [2–4].

Other appellations were used such as Stafne's idiopathic bone cavity, Stafne's bone cavity, Stafne's bone cyst, lingual mandibular salivary gland depression, lingual mandibular cortical defect, latent bone cyst, or static bone cyst [4].

It is generally accidentally discovered by intraoral or panoramic radiographs during dental treatments, and it is generally asymptomatic [2, 3].

Radiography illustrates this defect as a unilocular radiolucent image with a well-defined border and a regular border, found below the mandibular canal and can prolong from the molars to the angle [4].

Diagnosis is often made in panoramic radiographs only [5], but in some cases, computed tomography scanning (CT) that provides more precise images [6], cone-beam computed tomography (CBCT) with or without sialography [7, 8], and magnetic resonance imaging (MRI) are obligatory [8–10]. The SBD could be confused with cysts or tumors in the mandible [4].

## 2. Case Report

A 60-year-old male patient was oriented to our clinic for implant placement on the right mandible to restore missing first and second molars.

**Figure 1:**
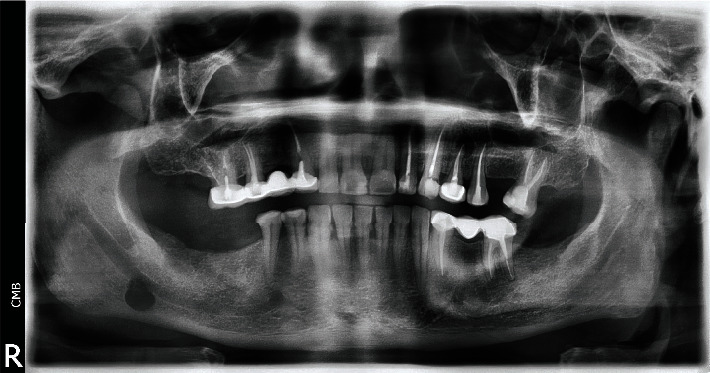
Panoramic radiograph showed a well-limited radiolucency image with a clear peripheral regular condensation border, located below the mandibular canal.

Panoramic radiograph revealed a well-limited radiolucency image with a clear peripheral regular condensation border, located below the mandibular canal (Figure 1).

No history of trauma of the jaw or pain or swelling was recorded by the patient. No facial asymmetry or lymphadenopathy was detected on clinical exams, and the third right mandibular molar was missing. At this stage, several diagnoses were possible, including traumatic bone cyst, residual cyst, dentigerous cyst, odontogenic keratocyst, focal osteoporotic bone marrow defect, and Stafne's bone defect.

For more evidence on the cavity and for final diagnosis, CBCT was recommended.

**Figure 2:**
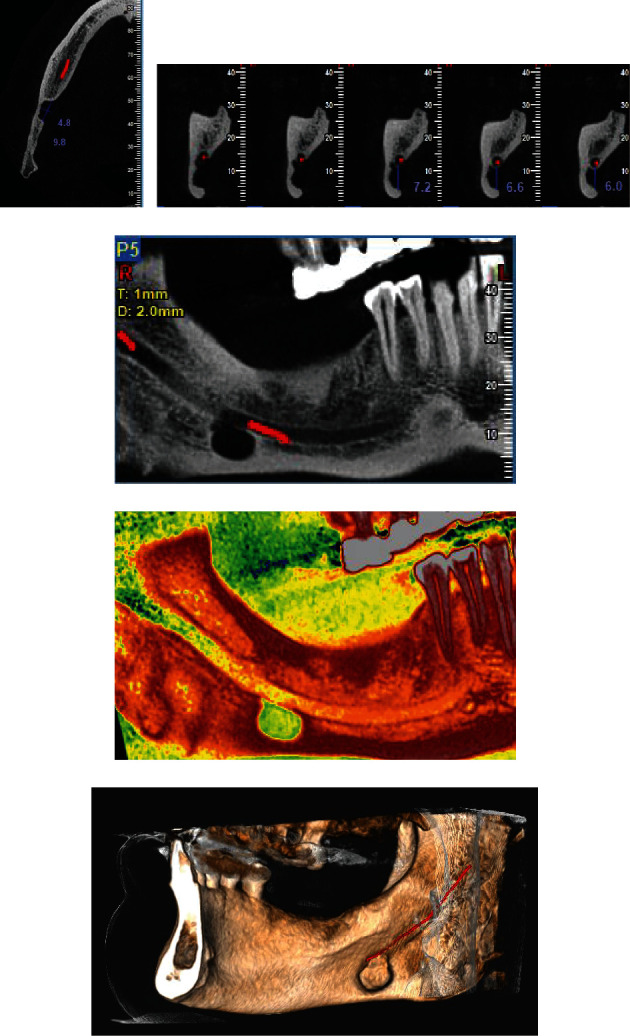
(a) The axial cuts of a CBCT showed a well-defined lingual defect (9.8 × 4.8 mm) with a thin cortical border in the posterior part of the right mandible. (b) The para-axial cuts revealed a cavity in the lingual side of the mandible that has a lingual opening, is monocystic, and has a cortical margin below the mandibular canal with ±7.2 mm distance. (c) Panoramic reconstruction offered a view of the lesion separated from the mandibular canal and well limited by a thin cortical. (d) Panoramic reconstruction with the visualization window spectrum color showing the borders of the bone defect and the relation with the mandibular canal. (e) The 3D reconstruction presented the shape, location of the well-limited defect filled with soft tissues, and its relation with the mandibular canal.

The axial cuts revealed a well-defined lingual deficiency with a thin cortical border in the posterior part of the right mandible with a dimension of 9.8 × 4.8 mm (Figure 2(a)).

The para-axial cuts revealed a monocystic defect in the lingual part of the mandible communicating with the submandibular area having a cortical margin below the mandibular canal with ±7.2 mm distance (Figure 2(b)).

The panoramic reconstruction offered a view of the lesion separated from the mandibular canal and well limited by a thin cortical (Figures 2(c) and 2(d)).

The 3D reconstruction presented the shape, location of the well-limited defect filled with soft tissues, and its relation with the mandibular canal (Figure 2(e)).

In order to diagnose the type of the soft tissue inside the bone defect, MRI was prescribed.

MR images were obtained on a 3T machine (SIGNA™ Architect 3.0T, 70 cm MRI scanner) using AIR™ 48ch Head Coil.

MRI of the neck performed in a 3 T ultraconductive field included 5 mm slice thickness coronal sections in short tau inversion recovery- (STIR-) weighted, axial T1-weighted spin echo (SE), axial and coronal T2-weighted fast spin echo (FSE), and T2-FSE with deletion of the fat signal.

**Figure 3:**
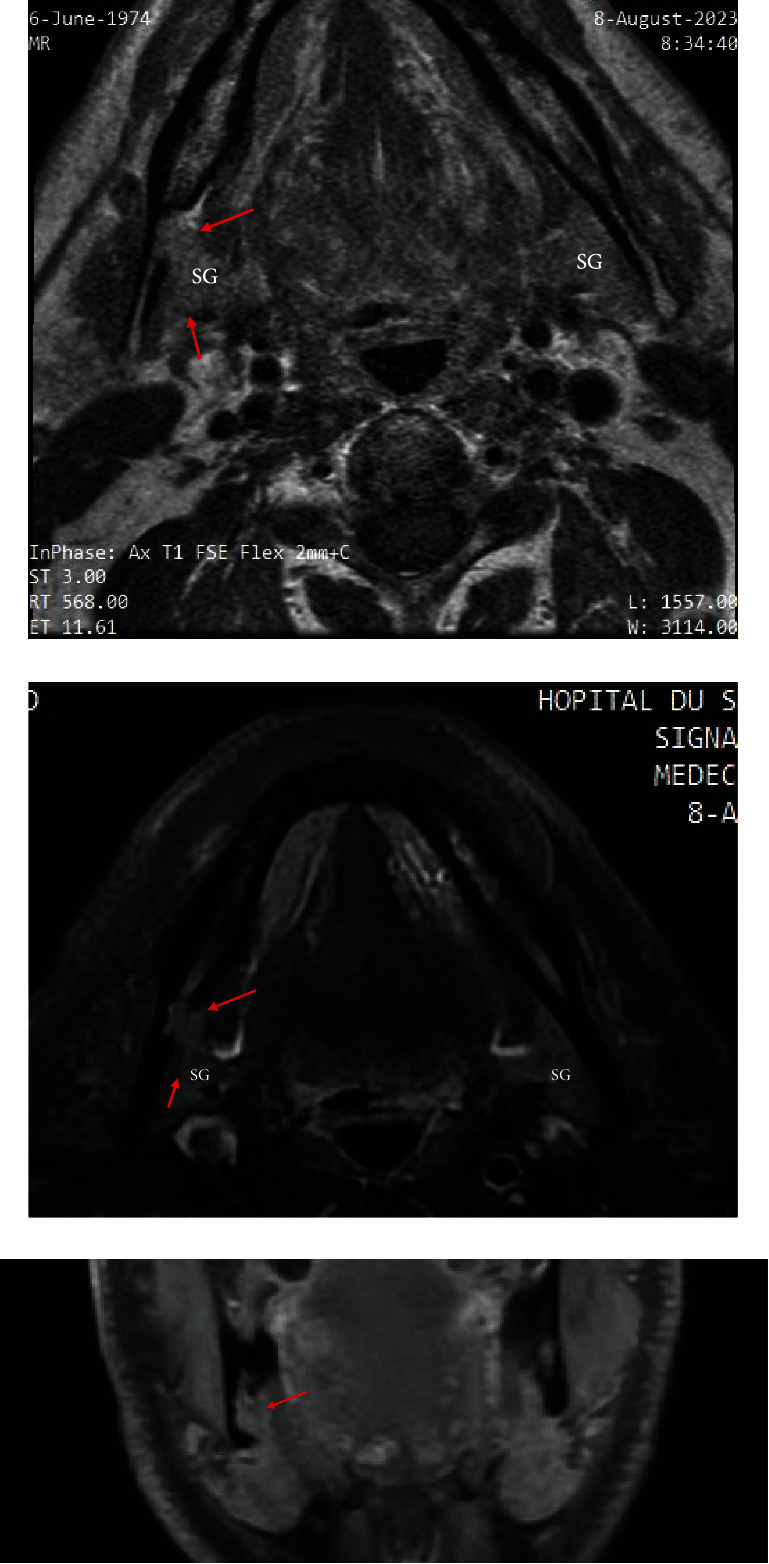
(a) Axial T1 FSE shows that the mandibular defect (red arrows) is filled with soft tissue in continuity with and has the same signal intensity with the submandibular gland (SG). The contralateral gland (SG) is marked for comparison. (b) The right mandible defect was again identified with the axial T2 FS, and it contains soft tissue that was continuous with the adjacent submandibular gland (red arrows) and was identical in signal intensity to the left gland (SG). (c) Coronal T1 FSE shows that the bony defect (red arrows) contains an extension of the submandibular gland.

The radiolucent lesion was ovoid and well demarcated with a thin cortical border lingually in the body of the mandible (Figure 3(a)).

MRI displayed that the bone cavity was filled with soft tissues and the insides of the cavity emanated the same MRI sign as the submandibular gland (Figure 3(b)).

MRI established that the cavity was in continuity with the submandibular gland (Figure 3(c)).

The diagnosis of SBC was reserved. No additional examinations or treatments were suggested.

## 3. Discussion

The etiology of SBD is still indeterminate, and the “glandular” hypothesis is the most common pathogenesis [4]. Following this theory, the submandibular or sublingual glands during their developments produce compression of the lingual part of the mandible, tracked by resorption of the cortical bone and ultimately developing a defect occupied with glandular tissue [4, 11–13].

Lello and Makek [14] proposed the “ischemic” theory. The defect is due to a relative ischemia that disturbs the vascularization of the mandibular lingual cortex [14].

Ariji et al. [15] classified SBD concavities according to their outline and relationship to the buccal cortical plate, based on CT scans, into three types: type I: the bottom of the defect is not reaching the buccal cortical bone; type II: it affected the buccal cortical bone, but there was no extension of the bone; and type III: it was characterized by a buccal expansion of the cortical plate.

Philipsen et al. [11] categorized SBD into three topographical variants: (1) above the mylohyoid muscle facing the lingual anterior mandibular body, (2) posterior to the mandibular angle below the mandibular canal, and (3) lingual side of the ramus below the neck of the condyle and posterior to the lingual foramen.

An exceptionally rare variant is located to the buccal part of the mandibular ramus [13].

Schneider et al. [12] identified 21 SBD (0.7%) among 2928 patients on panoramic radiographs. The male/female quotient was 14/7 with a mean age of 53 years. All defects were cited in the posterior part of the mandible, and the mean length was 10.9 mm and height 5.7 mm.

Assaf et al. [16] found only 11 cases of SBD (0.08%) after analyzing 14,005 panoramic radiographs with a male predilection, and the mean age was 58.1 years. In 8 cases only, SBD was located in the mandibular corpus and in 3 cases in the angle.

The SBD mandibular defects in the molar region in the mandible have been described in 19 cases (0.475%) after examining 4000 panoramic radiographs of Taiwanese patients. They have a suggestive male tendency with 90% [4].

Soares et al. [17] in a systematic review, where 465 patients with SBD were included, stated that the average age was 52.78 years, with male predilection (80.85%). Panoramic radiographs were the common radiological tests (64.09%), followed by CT (21.08%). SBD was more predominant in the posterior part of the mandible (93.77%) as radiolucent lesion (77.40%). Mean size was 1.58 cm.

Hayashi et al. [18] reported a case of an SBD extending from the mandibular anterior to the premolar region, and they found after a CT and MRI explorations that salivary gland tissue connected to the sublingual glands was involved in the formation of the cavity.

SBD can be eagerly detected with a panoramic X-ray due to their distinctive characters [4]. CBCT is the most frequently exam used as a noninvasive and accurate test for SBD diagnosis [19, 20].

CT has supplementary benefits on panoramic radiographs for the 2D and 3D visualization of the bone [6, 19, 20]. SBD is described, on CT, as a round defect, monocystic with a very well-defined cortical margin situated on mandibular lingual side [6, 19, 20]. SBD can be discovered when these characteristics are detected, and no supplementary exams are reflected required [19, 20].

CT with sialography is more beneficial than CT alone when exploring relations with the major salivary glands [7]. This invasive technique implicates cannulation of the salivary glands' ducts, by injection of contrast liquid and exposure to radiations [7, 20].

Some authors contemplated that CT is the most efficient exam for detecting SBD [19], while others have discovered that CT is lacking and MRI is the most applicable exam [9, 10, 20].

MRI with STIR-weighted coronal sections, T1-weighted, T1-weighted transverse axis, and T2-FSE revealed that the bone cavities were occupied with tissues comparable to the submandibular gland [20].

MRI indorsed to find a hyperintense signal for fluid structures with subtraction of adjacent tissue on T2-FSE with deletion of the fat signal and can thus stress the salivary ductal system; furthermore, MRI can envisage the bone marrow and the cortical [6, 8, 10, 20].

MRI allows to visualize the submandibular gland ducts and identifies the presence of salivary gland tissue inside cavities with accurate information, and this technique does not engage cannulation or injection and does not irradiate the patient [9, 10, 21].

The difference between CT and MRI is that there is no radiation exposure and has greater determination for illustrating the type of tissue filling the SBD [10, 20]. MRI indicates the normality of the tissue, and no more tests or surgical action will be needed [20].

Clinically, the SBD is mainly established on the symptomless radiolucent bone cavity which distinguishes it from other pathologies regardless of the content and location of the lesion [21].

The differential diagnosis of SBC involves traumatic bone cyst, radicular cyst, odontogenic cystic lesion, nonossifying fibroma, focal bone marrow defect related to osteoporotic process, brown tumor in hyperparathyroidism, and vascular malformation [22, 23]. Although posterior variants are frequently diagnosed on panoramic radiographs and variable localizations can be confused with inflammatory lesions or odontogenic cystic lesions, an avoidable treatment might be done [22, 23].

In our case, the localization of the SBD was in the posterior part of the mandible as described previously by Chen et al. [4], Hisatomi et al. [5], Schneider et al. [12], and Soares et al. [17]

The size of the reported SBD was 9.8 × 4.8 mm smaller than what was stated by some authors as 10.9 mm × 5.7 mm [12], 1.58 cm [17], and 7.9 mm × 16.3 mm [24].

The radiographs, in our case, revealed a well-limited radiolucency image with a clear peripheral regular condensation border, located below the mandibular canal in concordance with the type II of Ariji et al. [15] and the type 2 described by Philipsen et al. [11].

The shape and size of our lesion were discovered in a routine radiographic exam, the CBCT gave us more details about the localization and relation with the mandibular canal, and the MRI identifies the nature of the inside soft tissue.

The final diagnosis was an SBD resulting of a defect affecting the lingual cortical bone filled with expansion of the submandibular gland.

No further treatment was needed, and only observation within the time will be required.

## 4. Conclusion

SBD is a benign, developmental bony defect usually without any pathological disorders.

A case of Stafne's bone defect extending in the posterior part of the mandibular region was offered.

CBCT and MRI advised that salivary gland tissue linked to the submandibular gland was intricate in the development of the cavity. The presented case joins the etiology of “glandular” hypothesis.

Only regular radiographic follow-ups are suggested to control any enlargement tendency or any abnormal modifications of the lesion.

In conclusion, an association of CBCT and MRI was discovered to be a favourable methodology for exploring SBD. CBCT specified evidence about shape, size, and anatomical borders and MRI for the identification of the type of glandular tissue in the defect and endorsed a final diagnosis.

## Data Availability

All available data is in the manuscript.
